# Activation of the Cell Wall Stress Response in *Pseudomonas aeruginosa* Infected by a Pf4 Phage Variant

**DOI:** 10.3390/microorganisms8111700

**Published:** 2020-10-30

**Authors:** Damien Tortuel, Ali Tahrioui, Sophie Rodrigues, Mélyssa Cambronel, Amine M. Boukerb, Olivier Maillot, Julien Verdon, Emile Bere, Michael Nusser, Gerald Brenner-Weiss, Audrey David, Onyedikachi Cecil Azuama, Marc G. J. Feuilloley, Nicole Orange, Olivier Lesouhaitier, Pierre Cornelis, Sylvie Chevalier, Emeline Bouffartigues

**Affiliations:** 1 Laboratoire de Microbiologie Signaux et Microenvironnement, Université de Rouen Normandie, Normandie Université, LMSM EA4312 Évreux, France; damien.tortuel@etu.univ-rouen.fr (D.T.); ali.tahrioui@univ-rouen.fr (A.T.); sophie.rodrigues@univ-ubs.fr (S.R.); melyssa.cambronel@etu.univ-rouen.fr (M.C.); amine.boukerb@univ-rouen.fr (A.M.B.); olivier.maillot@univ-rouen.fr (O.M.); audrey.david5@univ-rouen.fr (A.D.); onyedikachi.azuama@etu.univ-rouen.fr (O.C.A.); marc.feuilloley@univ-rouen.fr (M.G.J.F.); nicole.orange@univ-rouen.fr (N.O.); olivier.lesouhaitier@univ-rouen.fr (O.L.); pcornel@vub.ac.be (P.C.); 2 Laboratoire Ecologie et Biologie des Interactions, Université de Poitiers, UMR CNRS 7267 Poitiers, France; julien.verdon@univ-poitiers.fr (J.V.); ebere@univ-poitiers.fr (E.B.); 3 Institute of Functional Interfaces, Karlsruhe Institute of Technology, 76131 Karlsruhe, Germany; michael.nusser@kit.edu (M.N.); gerald.brenner-weiss@kit.edu (G.B.-W.)

**Keywords:** *Pseudomonas aeruginosa*, Pf4 phage variant, SigX, AlgU, cell envelope, biofilm, c-di-GMP, membrane fluidity, cell wall stress response

## Abstract

*Pseudomonas aeruginosa* PAO1 has an integrated Pf4 prophage in its genome, encoding a relatively well-characterized filamentous phage, which contributes to the bacterial biofilm organization and maturation. Pf4 variants are considered as superinfectives when they can re-infect and kill the prophage-carrying host. Herein, the response of *P. aeruginosa* H103 to Pf4 variant infection was investigated. This phage variant caused partial lysis of the bacterial population and modulated H103 physiology. We show by confocal laser scanning microscopy that a Pf4 variant-infection altered *P. aeruginosa* H103 biofilm architecture either in static or dynamic conditions. Interestingly, in the latter condition, numerous cells displayed a filamentous morphology, suggesting a link between this phenotype and flow-related forces. In addition, Pf4 variant-infection resulted in cell envelope stress response, mostly mediated by the AlgU and SigX extracytoplasmic function sigma factors (ECFσ). AlgU and SigX involvement may account, at least partly, for the enhanced expression level of genes involved in the biosynthesis pathways of two matrix exopolysaccharides (Pel and alginates) and bis-(3′-5′)-cyclic dimeric guanosine monophosphate (c-di-GMP) metabolism.

## 1. Introduction

*Pseudomonas aeruginosa* is a ubiquitous Gram-negative γ proteobacterium that thrives in different environments, including soils, water, nematodes, plants and mammals [1,2]. This opportunistic pathogen is responsible for a wide range of infections in the lungs, urinary tract, and burn wounds [3] *P. aeruginosa* can form multi-cellular matrix-enclosed biofilms [4] that protect cells from phagocytosis [5] and contribute to antibiotics resistance, and its persistence in lung infections [6].

Filamentous phages (Inoviridae) are widespread in the microbial world. They have a long and thin filamentous shape and contain a single-stranded circular DNA genome [7,8]. In Gram-negative bacteria, inoviruses are thought to be mostly lysogens with the phage genome either integrated into the bacterial chromosome or being present as an extrachromosomal episome [7]. *P. aeruginosa* strains integrate in their genome several filamentous bacteriophages designated as Pf phages. *P. aeruginosa* PAO1 contains only Pf4. Phage Pf4 is closely related to Pf5 of *P. aeruginosa* PA14, and to a lesser extent to Pf1 of PAK strain [7]. While Pf4 and Pf5 are maintained as prophages, Pf1 replicates exclusively as an episome [9]. The pathway of infection of different bacteria by filamentous bacteriophages is conserved through binding to a host-specific pilin receptor [10]. Hence, bacteriophages Pf1 and Pf4 use as receptors type IV pili (TFP) from PAK [11] and PAO1 [12,13], respectively, while Pf3 of strain PAO binds to the RP4 conjugative pilus [11].

Clinical data show that Pf phages are produced by *P. aeruginosa* isolates from sputum of cystic fibrosis (CF) suffering patients [14,15,16] at an average 10^7^ copies of Pf phage.mL^−1^ sputum [17,18]. Increasing amounts of evidence suggest that filamentous phages can strongly affect biofilm maturation and dispersal [14,19]. Pf4 was indeed shown to be involved in matrix structuration [17], and to increase cell lysis leading to extracellular DNA (eDNA) release into biofilm matrix [14,20,21,22]. In addition, Pf4 infection can lead to the emergence of small colony variants [14,18]. Deletion of the whole prophage genomic region results in less-stable biofilm when challenged with the sodium dodecyl sulfate detergent [19]. Pf4 genes are among the most highly induced during biofilm formation compared to planktonic growth. The same genes were found upregulated under anaerobic growth condition at pH 6.5 in the presence of nitrate or nitrite as electron acceptors [16,20]. Pf4 proteins were also overproduced in response to solid surface attachment [23]. Interestingly, biofilm maturation is related to the conversion of Pf4 phage into a superinfective variant [24]. While the lysogenic *P. aeruginosa* PAO1 strain carrying the Pf4 prophage integrated into its chromosome is resistant to Pf4 phage infection [19], the same lysogenic *P. aeruginosa* strain becomes relatively sensitive to an infection by the superinfective variant phage, which can partly lyse the prophage-carrying host [25]. Noticeably, this Pf4 variant is characterized by several mutations, which are mostly located within or upstream of *repC*, a Pf4-encoded repressor involved in the active life cycle of the phage [13], recently renamed *pf4r* [26]. Such mutations have been suggested to result from DNA lesions in response to reactive oxygen or nitrogen species accumulation during growth in biofilm [24]. Moreover, it has been demonstrated that the OxyR oxidative stress response regulator binds to a region overlapping the *pf4r* open reading frame [24,27]. Accordingly, the oxidative stress response mediated by OxyR has been correlated to the superinfective variant phage emergence in biofilm [24]. In addition, Pf4 superinfection would also be observed when a high titer of wild-type Pf4 overwhelm superinfection exclusion mechanism in *P. aeruginosa* PAO1 [28].

In *P. aeruginosa*, cell wall integrity alterations induce a cell envelope stress response (CESR), which reprograms gene expression to ensure bacterial survival via two extracytoplasmic function (ECF) σ factors, AlgU and SigX [29]. AlgU is homologous to the *Escherichia coli* RpoE CESR σ factor that is mainly involved in outer membrane and peptidoglycan homeostasis [30,31]. AlgU is activated in response to hyperosmolarity [32], peptidoglycan synthesis alterations [30,31] or cell wall perturbations related to absence of the major porin OprF [33]. Expression of *oprF* is controlled by AlgU [34] and SigX [35,36], and a mutant lacking OprF shows increased expression and activity of SigX in addition to AlgU [33]. SigX is activated in response to hypo-osmolarity [35,37], high sucrose concentration [38], cold-shock condition or valinomycin treatment [39]. As SigX is involved in de novo fatty acid biosynthesis [40,41], SigX activity affects membrane homeostasis [37,42,43] and membrane fluidity [29,40,41]. In addition to their role in maintaining *P. aeruginosa* peptidoglycan and membrane homeostasis, AlgU and SigX are also involved in motility, biofilm formation and virulence [29]. This study aims to obtain further insights into the physiology of *P. aeruginosa* H103 (the PAO1 strain originating from Hancock’s laboratory [44] after infection by a Pf4 filamentous phage variant, using combined phenotypic and gene expression analyses.

## 2. Materials and Methods

### 2.1. Pf4 Phage Variant Production

A library of *P. aeruginosa* H103 mutants was created by random transposon mutagenesis to study the transcriptional regulation of *sigX* (data not shown). The mini-CTX-lux plasmid with the promoter region of *sigX* was integrated into the *attB* site in *P. aeruginosa* H103 resulting in dH103 strain (Supplementary Table S1). The screening of the library allowed the identification of dH103Pf4^+^ mutant (Supplementary Table S1), which displayed a partial cell lysis phenotype and overproduced Pf4 phage variant that were named Pf4* in the following sections. To obtain Pf4* phages, the mutant dH103Pf4^+^ was grown for 24 h at 37 °C, then, 1 mL of the planktonic culture was harvested and centrifuged at 8000× *g* for 5 min. The supernatant was then filtered using a 0.22 µm pore size filter and stored at 4 °C until further use.

### 2.2. Bacterial Strains, Media, and Growth Conditions

Bacterial strains used in this study are listed in Supplementary Table S1. For planktonic cultures, bacteria were inoculated in LB medium containing 50 mM NaCl at an initial absorbance at 580 nm (A_580 nm_) of 0.08. To perform RNA extraction, bis-(3′-5′)-cyclic dimeric guanosine monophosphate (c-di-GMP) and anisotropy assays, bacteria were grown at 37 °C with orbital shaking at 180 r.p.m for 5 h (wild-type) or 7 h (Pf4*-T) to reach an A_580 nm_~2.8. For infection experiments, supernatant of dH103Pf4^+^ containing Pf4 phage variants, and lytic LUZ19 phages [45], were added to the planktonic cultures at 1.5 × 10^9^ phages.mL^−1^ (final concentration). Supernatant containing Pf4* phages was added at the beginning of the cultures (A_580nm_ = 0.08) for all experiments and LUZ19 phages, when used to observe cell killing, were added at the middle of exponential growth (A_580 nm_ = 0.5). For co-infection experiments, supernatant containing Pf4 phage variants was added at the beginning of the culture (A_580 nm_ = 0.08), and LUZ19 phages were added after 3 h of growth (end of the exponential growth (A_580 nm_ = 2)) in order to saturate Pf4* receptors of H103. All experiments were performed independently at least four times.

### 2.3. Numeration of Pf4* Phages by Double Agar Overlay Plaque Assay

The double agar overlay plaque assay was performed as previously described [46] with some modifications. Briefly, the bottom agar was prepared by adding 15 g.L^−1^ technical agar (Becton Dickinson, Franklin Lakes, NJ, USA) to LB broth medium, sterilized and dispensed in Petri dish plates. The top agar was prepared by dissolving 6 g.L^−1^ noble agar (Becton Dickinson, Franklin Lakes, NJ, USA) in distilled water, sterilized and kept at 60 ºC in a water bath. Pf4* phages from the supernatant were diluted by tenfold serial dilution in LB broth medium. Then, 10 µL of each dilution and planktonic cultures used as receptor strains (H103 or PAK) adjusted at A_580 nm_ = 0.1 were added to 5 mL of top agar. The overlay mixture was next dispensed on the bottom agar and the plates were incubated at 37 °C for 24 h. A supernatant stock solution obtained from planktonic cultures of H103 strain following the same protocol as described above (see Pf4* phages production section) was used as a control condition.

### 2.4. Quantitative Reverse-Transcription Real-Time PCR (RT-qPCR) Analyses

Total RNAs were extracted from phage-infected *P. aeruginosa* cultures using the hot acid-phenol method [35]. Genomic DNA contamination was removed by using rigorous treatment with RNAse-free Turbo DNaseI kit (Invitrogen, Carlsbad, CA, USA), following manufacturer’s instructions. The concentration of RNAs was determined using the NanoDrop ND-2000 spectrophotometer (ThermoFisher Scientific, Waltham, MA, USA) and their quality was checked on an agarose gel (2%). RNAs were converted to cDNA by the High-Capacity cDNA Reverse Transcription kits (Applied Biosystems, Foster City, CA, USA), according to manufacturer’s recommendations. Quantitative PCR reactions were carried out using SYBR Green PCR Master Mix™ 335 (Applied Biosystems, Foster City, CA, USA) in an ABI 7500 Fast Q-PCR system (Applied Biosystems, Foster City, CA, USA). All primers used in this study were designed with Primer3 plus software (Supplementary Table S2) and were chemically synthesized and purified by Eurogentec (Liège, Belgium). The mRNAs expression levels were normalized on 16 S rRNA threshold cycle (Ct) values and calculated by comparing the Ct of targeted genes with those of control sample groups. The relative quantification data were determined with the 2^−ΔΔCt^ method using Microsoft Excel-based spreadsheet.

### 2.5. Twitching Motility

Planktonic cultures of H103 treated or untreated with Pf4* phage in LB medium were assayed for twitching motility. An aliquot of 5 µL diluted to an A_580 nm_ of 0.1 was used to inoculate the semi-solid LB medium agar plates (1% agar *w/v*) underneath the agar layer. After 48 h of incubation at 37 °C, the agar was removed and the bacterial cells that adhered to the Petri dish were directly stained with 0.4% crystal violet for 15 min and washed with distilled water, as previously described [43]. Then, representative pictures were taken.

### 2.6. Biofilm Culture

Biofilm formation under static conditions was performed in 24-well plates with flat glass bottoms (Greiner Bio-One, Kremsmünster, Austria). Bacterial cells of H103 strain were harvested from overnight cultures by centrifugation (7500× *g*, 5 min) and washed twice with sterile physiological solution (9 g.L^−1^ NaCl). Prior to biofilm formation, an adhesion step was carried out by inoculating each well with a bacterial suspension adjusted to an A_580 nm_ of 0.1 in sterile physiological solution for 2 h at 37 °C. Non-adhered planktonic cells were then removed and LB medium at 50 mM NaCl with or without Pf4* phages was added and biofilm cultures were grown at 37 °C for 22 h. Biofilms were washed twice by sterile physiological solution. Biofilm formation was also examined under hydrodynamic conditions, as described by Rodrigues and colleagues [47], in a three-channel flow cell with individual channel dimensions of 1 mm × 4 mm × 40 mm (Biocentrum, Kraków, Malopolskie, Poland) [48], using a microscope glass coverslip (24 × 50 Knittel Glass, Varrentrappstraße, Braunschweig, Germany) as a substratum. The flow system was prepared and sterilized as previously described [49]. Briefly, sterilization was performed by 0.5% sodium hypochlorite for 30 min and rinsed overnight by sterile physiological solution using a 250 S Watson Marlow peristaltic pump. Bacteria were harvested from overnight culture by centrifugation (7500× *g*, 5 min), washed twice with sterile physiological solution and adjusted at _A580 nm_ = 0.1. Bacteria were allowed to adhere for 2 h at 37 °C without flow. Then, LB medium at 50 mM NaCl with or without Pf4* phages was pumped with a 2.5 mL.h^−1^ flow for 22 h at 37 °C.

### 2.7. Confocal Laser Scanning Microscopy (CLSM)

Prior to CLSM examination, bacteria were stained with 5 µM of SYTO9 Green 5 dye (Invitrogen, Carlsbad, CA, USA) for 15 min. Static biofilms were then washed twice with sterile physiological solution and dynamic biofilms were rinsed for an additional 10 min with a flow of medium. Biofilms were observed with a Zeiss LSM710 microscope (Carl Zeiss Microscopy, Oberkochen, Germany) using a 40× oil immersion objective. SYTO9 was excited at 488 nm and fluorescence emission was detected from 500 to 550 nm. Images were recorded every micrometer, visualized, and processed using the Zen 2.1 SP1 software (Carl Zeiss Microscopy, Oberkochen, Germany). Quantitative analyses of 3D images (thicknesses and biovolumes) were performed using COMSTAT2 software (http://www.imageanalysis.dk/) [50,51]. At least three image stacks from each of four independent experiments (twelve stacks in total) were used for each analysis.

### 2.8. Transmission Electron Microscopy (TEM)

Pf4* phages were resuspended in PBS and a drop of suspension was placed on a petri dish. A Formvar EM grid was placed Formvar side down on top of the phages drop for approximately 1 min. The grid was removed, blotted with filter paper, and placed onto a drop of 7.0% uranyl acetate in distilled water for 3 min. The grid was washed in water, blotted again with filter paper, and examined on EM JEOL 1010 at 80 KV. The pictures were recorded by using Quemesa, the electron digital camera of Olympus.

### 2.9. c-di-GMP Quantification

c-di-GMP levels were quantified by LC-MS/MS as previously described [33,52]. H103 cells treated or untreated by Pf4* phage (Pf4*-T) were harvested, centrifuged (8000× *g*, 2 min, 4 °C), and washed in sterile physiological solution (0.9 g.L^−1^ NaCl). Then, bacterial pellets were resuspended in the extraction buffer (acetonitrile/methanol/ultrapure water (2V/2V/1V)) and incubated for 15 min on ice. Next, bacteria were lysed at 95 °C for 10 min and centrifuged at 20,000× *g* for 5 min at 4 °C. The supernatants were conserved on ice and the extraction was repeated twice. All supernatants were pooled together and evaporated at 43 °C for 24 h. After resuspension in 400 µL of ultrapure water, the samples were subjected to direct quantification by LC-MS/MS. The chromatographic separation was performed on a 1100 Series HPLC system (Agilent, Waldbronn, Germany) using a Multospher AQ RP18, 5 µm, 250 mm × 4.0 mm HPLC column (CS Chromatography Service GmbH, Langerwehe, Germany) in a gradient mode using methanol as eluent A and 10 mM ammonium acetate and 0.1% acetic acid in ultrapure water as eluent B. The injection volume of each sample and the flow rate were set at 40 μL and 0.40 mL.min^−1^, respectively. The gradient program was as follows: from 0 to 4 min 100% B, followed by a linear gradient from 100 to 80% B in 22 min, held for 2 min at 80% B, followed by a linear gradient from 80 to 60% B in 1 min and held for an additional 9 min at 60% B. Finally, re-equilibration of the column was obtained by constantly running 100% B for 16 min. Electrospray ionization (ESI) MS was performed on an API 4000 triple quadrupole mass spectrometer (Applied Biosystems, Toronto, ON, Canada) using a turbo ion spray interface in positive mode at an ionization potential of 4800 V and a temperature of 450 °C. Nitrogen gas was used as curtain, nebulizer, heater and collision gas. The parameter settings were optimized by infusion experiments. Data were acquired in multiple reaction monitoring modes (MRM) using the Analyst software version 1.6.3 (Applied Biosystems, Toronto, ON, Canada). Identification and quantification of c-di-GMP were performed by using three specific mass transitions from molecule ion *m/z* 691 to the product ions 152, 135, and *m/z* 540. The external calibration was carried out at c-di-GMP concentrations ranging from 1 to 100 ng.mL^−1^ (ultrapure water), analyzed in duplicate.

### 2.10. Membrane Fluidity Assays by Fluorescence Anisotropy

Fluorescence anisotropy measurements were performed as previously described [41,53]. *P. aeruginosa* H103 cells treated or untreated with Pf4* phage (Pf4*-T) were harvested by centrifugation (8000× *g*), washed twice in sterile Tris/HCl buffer (15 mM, pH 7), and resuspended in the same buffer to reach an A_580 nm_ = 0.2. Then, 1 µL of a 4 mM of 1,6-diphenyl-1,3,5-hexatriene (DPH, Sigma Aldrich, Saint-Louis, MI, USA) stock solution in tetrahydrofuran was added to 1 mL aliquot of the resuspended cells and incubated in the dark for 30 min at 37 °C to allow the probe to incorporate into the cytoplasmic membrane. Further, anisotropy measurements were performed using the Spark^®^ 20 M multimode microplate reader (Tecan, Männedorf, Switzerland), equipped with an active temperature regulation system. Excitation and emission wavelengths were set at 365 and 425 nm, respectively, with a gain of 95. The G correlation factor was calculated as the relation between the horizontal and vertical emitted light after the excitation by a horizontal light. In our conditions, the G factor was determined to 1.596. The anisotropy was calculated from emission fluorescence intensity measured alongside (*I*1) and perpendicularly (*I*2) to light excitation plan, according to the equation:

$$r=\frac{\left(I1-I2\right)}{\left(I1+2\;GI2\right)}$$

The relationship between anisotropy and membrane fluidity is an inverse one, where decreasing anisotropy values correspond to a more fluid membrane and vice versa.

### 2.11. Pf4 Prophage Region Sequencing

Bacterial DNA was extracted from dH103Pf4^+^ and H103 overnight cultures using GeneJet Genomic DNA extraction kit (ThermoScientific, Waltham, MA, USA). A genomic library for whole genome sequencing was prepared using the Nextera^TM^ XT DNA library prep kit (Illumina, San Diego, California, USA). Sequencing was performed on an Illumina MiSeq platform (LMSM Evreux, Rouen Normandy University, France) using a paired-end protocol (2 × 250 bp). Raw paired-end reads were trimmed using Trimmomatic v.0.36 [54]. Sequence data quality was checked using FastQC v.0.11.6 [55]. Assembly of trimmed paired-end reads was done de novo using Unicycler v.0.4.7 [56]. The obtained draft was checked for consistency, e.g., the number of contigs, G + C content and total size of assembly, using Quast v.5.0.0 [57]. The extracted phage sequence from *P. aeruginosa* PAO1 (Genbank accession number: GCA_000006765.1) was mapped to the trimmed raw reads with BBmap v38.69 from BBTools package [58]. Mapped reads in SAM format were converted into fastq files using SamToFastq command in Picard (https://broadinstitute.github.io/picard/). The obtained reads covering the phage region were mapped to PAO1 and H103 reference genomes using Snippy v.4.3.6 (https://github.com/tseemann/snippy) to detect SNPs or deletions, and assembled into contigs as described above. Default parameters were used except where otherwise noted.

### 2.12. Promoter Research

Promoters upstream of the *mreB* gene were predicted with BPROM (www.softberry.com) and by visual inspection, from *P. aeruginosa* PAO1 genome [59] (www.pseudomonas.com).

### 2.13. Statistical Analyses

The data were statistically analyzed using two-samples unpaired (for dH103 and dH103Pf4^+^ analysis) and paired two-tailed *t*-test to calculate *p* values with GraphPad Prism (GraphPad Prism version 4.0; GraphPad Software, San Diego, CA, USA). All values are reported and plotted as means with SEM of at least triplicate analyses for each experimental variable. NS (not significant); *p* > 0.05; * *p* < 0.05; ** *p* < 0.01; *** *p* < 0.001; **** *p* < 0.0001.

## 3. Results and Discussion

### 3.1. Production of Superinfective Pf4 Phages Variant under Planktonic Culture Conditions

**Figure 1:**
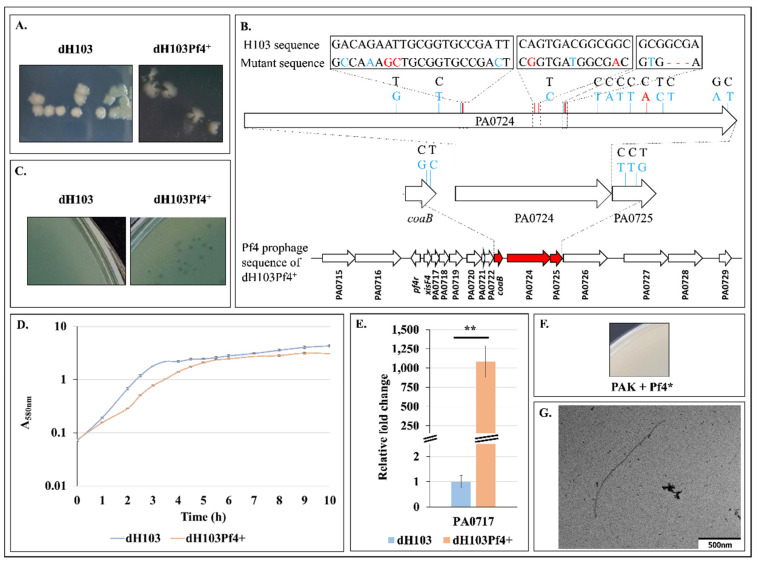
Isolation and description of a Pf4 phage variant. (**A**). Colony morphology of *P. aeruginosa* dH103 and dH103Pf4^+^ strains. (**B**). Gene mapping of the *coaB*-PA0725 Pf4 prophage region in dH103Pf4^+^ with mutations compared to H103. Silent mutations are represented in blue. Mutations and deletion are represented in red. (**C**). Lysis plaque assay using supernatant of dH103 and dH103Pf4^+^ performed on H103 strain. (**D**). Growth curves of dH103 and dH103Pf4^+^ ± SEM. (**E**). Relative mRNA levels of PA0717 in dH103 (blue bar) and dH103Pf4^+^ (orange bar) ± SEM as determined by RT-qPCR experiments. (**F**). Lysis plaque assay using supernatant of dH103Pf4^+^ performed on PAK strain. (**G**). TEM picture of Pf4* phage. Lysis plaques, growth and RT-qPCR experiments were assayed at least four times independently. Statistics were achieved by unpaired (two samples) two-tailed *t*-test. ** *p* < 0.01.

The screening of a transposon mutant library generated for another purpose (data not shown) led to the identification of dH103Pf4^+^, a transposon mutant derived from dH103 (see Materials and Methods section), displaying a colony lysis phenotype (Figure 1A). Genome sequencing of this mutant allowed identifying PA4722, encoding a probable aminotransferase (http://www.pseudomonas.com) as transposon insertion site. In addition, numerous mutations were identified in three genes belonging to the Pf4 prophage encoding region (red arrows in Figure 1B, Supplementary Figure S1). Most of these mutations are silent (Figure 1B, in blue), and include substitutions in PA0723 (*coaB*), PA0724 and PA0725, as well as a deletion in PA0724. Interestingly, only a few substitutions in PA0724 led to an amino acid change and the codon deletion resulted in one amino acid deletion (Figure 1B, in red; Table S3). Mutations in the genomic region encoding Pf4 prophage (PA0715-PA0729) are quite common in biofilm-grown *P. aeruginosa*, since these phages (qualified as superinfectives) are suggested to contribute to biofilm development and small colony variant formation [19]. However, to date, most of the described mutations have been localized in the *pf4r* gene or in the intergenic region between *pf4r* and *xisF4* (both genes are located within the intergenic region between PA0716 and PA0717). While Pf4r confers immunity to Pf4 by repressing active life cycle, *xisF4* product was recently identified as an excisionase promoting phage excision and activating phage replication, controlling the Pf4 switch between lysogeny and Pf4 production stage [26]. Moreover, superinfective Pf4 variants are able to form plaques on the wild-type host, which is otherwise resistant to reinfection by the non-superinfective phages [19]. Accordingly, the Pf4 variant isolated in the current study was able to induce lysis on its host lawns (Figure 1C) and affected the growth of its host since the generation time was increased from 37 min for dH103 to 57 min for dH103Pf4^+^ (Figure 1D). A Pf4 variant titer of about 4 × 10^10^ PFU.mL^−1^ was determined after 24 h of growth, which is in agreement with a previous study [60]. Overexpression of PA0717 by more than 1000-fold in dH103Pf4^+^ suggests that Pf4 phage variant overproduction might either be due to Pf4 variant multiplication/expression and/or from prophage expression (Figure 1E). Thus, a superinfective Pf4 phage variant was obtained herein from a planktonic and not from a biofilm culture. Since the supernatant collected from dH103Pf4^+^ strain might contain R2-type pyocin, we next assayed infection of *P. aeruginosa* by this supernatant. Lysis plaque assay using supernatant of dH103Pf4^+^ was then tested on *P. aeruginosa* PAK strain, which is known to be sensitive to pyocin R2 [61] but not to Pf4 phage [62]. No lysis plaque was observed on PAK strain, confirming the absence of R2-type pyocins in dH103Pf4^+^ supernatant (Figure 1F).

Since lysogenic *P. aeruginosa* strains are resistant to reinfection by non-superinfective phages [19], and because the Pf4 phage variants produced by dH103Pf4^+^ mutant strain displayed interesting phenotypes, we decided to use this variant to study the effects of such phage variant infection on *P. aeruginosa* H103 molecular and physiological responses. However, to date, the reasons behind the involvement of any mutation (including the insertion of the transposon within PA4722) in such variant overproduction by dH103Pf4^+^ strain, remain unexplained, and should be explored in forthcoming studies. Pf4 phage variant from dH103Pf4^+^ supernatant was thus used in the subsequent assays and was referred to as Pf4 variant or Pf4* in the following sections.

### 3.2. H103 Infection by the Pf4 Variant

**Figure 2:**
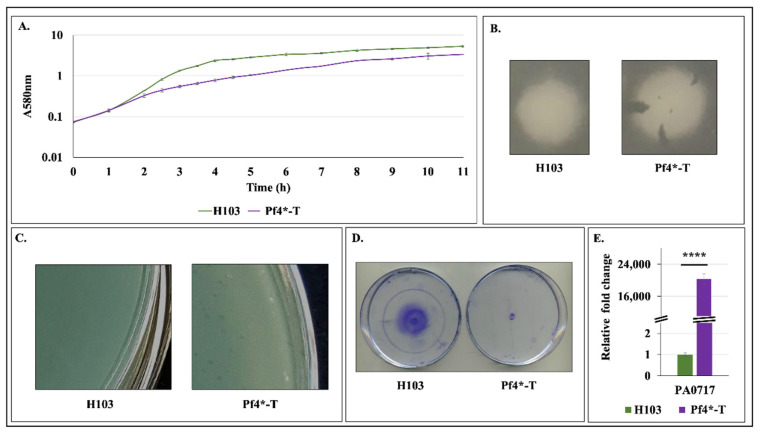
Effect of Pf4 phage variant on *P. aeruginosa* H103 strain. (**A**). Growth curves of H103 control strain and Pf4*-treated H103 (Pf4*-T) ± SEM. (**B**). Colony morphology of H103 and Pf4*-T. (**C**). Lysis plaque assay using supernatants of H103 and Pf4*-T strains performed on H103. (**D**). Twitching motility of P. aeruginosa H103 treated or untreated by Pf4* phages. (**E**). Relative mRNA levels of PA0717 in H103 (green bar) and Pf4*-T (violet bar) ± SEM as determined by RT-qPCR experiments. Growth, lysis plaques, twitching motility and RT-qPCR experiments were assayed at least four times independently. Statistics were achieved by paired (two samples) two-tailed *t*-test. **** *p* < 0.0001.

*P. aeruginosa* H103 infection was performed by adding dH103Pf4^+^ supernatant containing 1.5 × 10^9^ Pf4* PFU.mL^−1^ (final titer) at the beginning of the bacterial culture. As shown in Figure 2A, growth of Pf4*-treated (Pf4*-T) H103 was affected compared to H103, since the generation time was increased from 37 min (H103) to 55 min (Pf4*-T), in line with the data observed for dH103 and dH103Pf4^+^ strains (compare Figure 1D and Figure 2A). When plated on LB agar medium, Pf4*-T H103 displayed an altered colony morphology phenotype similar to the one of the dH103Pf4^+^ Pf4* phage producing strain (Figure 1A and Figure 2B). Moreover, Pf4 phage variant was able to form lysis plaques on *P. aeruginosa* H103 (Figure 2C). This result was quite surprising as the Pf4 phage was previously shown to be unable to form plaques on the wild-type PAO1 carrying the Pf4 prophage, while forming plaques only when the prophage is deleted from the genome [19]. These data suggest that the Pf4 phage variant produced by dH103Pf4^+^ can infect the wild-type strain H103 and thus resembling a superinfection. In addition, twitching motility, which relies on functional TFP, was totally abrogated in Pf4*-T condition (Figure 2E), in agreement with previous reports [62]. Expression of the PA0717 gene showed a huge increase of about 20,000-fold when *P. aeruginosa* was infected by Pf4* (Figure 2D), suggesting an active Pf4 or Pf4* replication in H103 strain. Thus, it is possible that the high titer of “wild-type” Pf4 (as suggested by PA0717 expression increase) will be sufficient to overwhelm any superinfection exclusion mechanism, as previously shown [26]. With these cautions in mind, Pf4* phage variant infection may be considered as resembling superinfection.

### 3.3. Pf4 Variant Infection Affects Biofilm Architecture and Cell Morphology

**Figure 3:**
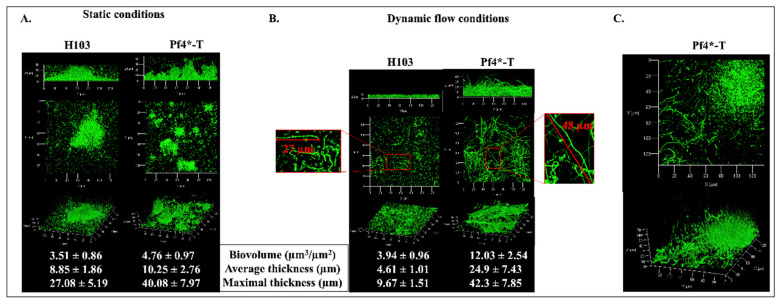
Pf4 phage variant infection leads to altered biofilm architecture and cell morphology. Side, top and three-dimensional views of 24 h-old biofilm of H103 untreated and treated with Pf4* phage grown under static (**A**) and dynamic conditions (**B**,**C**) observed by confocal laser scanning microscopy (CLSM). Cells were stained with SYTO9 green fluorescent dye. Values represent COMSTAT2 image analyses. Results were given ± SEM of at least three independent experiments. (**C**) Focus on microcolonies of the Pf4*-T biofilm grown in dynamic conditions. Each experiment was assayed at least four times independently.

So far, most studies have focused on mature biofilms, at least partly because such conditions were found to produce the superinfective Pf4 form [24]. Herein, the effect of Pf4 phage variant on *P. aeruginosa* H103 premature biofilm development was assessed using confocal laser scanning microscopy (CLSM) under both static (Figure 3A) and dynamic (flow cell, Figure 3B) conditions after 24 h of growth. *P. aeruginosa* H103 biofilm grown in static conditions displayed some mushroom-like structures (Figure 3A). Addition of Pf4* at the onset of the biofilm culture increased the number of these structures that appeared; however, they were smaller and scattered. COMSTAT2 image analyses indicated that Pf4*-T condition led to slightly higher maximal and average thicknesses, while the biofilm biovolume was not strongly affected compared to H103 (Figure 3A). Under dynamic flow conditions (2.5 mL.h^−1^), *P. aeruginosa* H103 displayed a flat and homogeneous biofilm architecture, mainly devoid of mushroom-like structures, with maximal and average thicknesses that were lower than when grown in static conditions (Figure 3B). COMSTAT2 image analyses indicated that only the maximum thickness was significantly increased (data not shown) in both cases. Noticeably, some bacteria were found to form chains, suggesting active multiplication of the resident bacteria that remain closely associated. Since such phenotype was not observed in static conditions, one can assume that it may result from the medium flow condition. The addition of Pf4* phage resulted in increased biofilm biovolume and both maximal and average thickness. Moreover, the cell morphology was strongly altered, since numerous single and extremely elongated thin cells were observed, with some of them reaching more than 40 µm in length. These filamentous structures were mostly located within the flat biofilm structure (Figure 3B). However, some rare microcolonies were observed, in which bacteria were displaying conventional length (1 to 2 µm), while the cells surrounding the mushroom-like structures were mostly extremely elongated (Figure 3C). The results indicate that filamentation/elongated H103 cell phenotype can be associated mainly with the presence of Pf4* phage under dynamic biofilm formation, possibly in response to general differences between the two conditions as nutrient supply, oxygenation or flow-related forces. A similar elongated-cell phenotype has been previously reported in response to major stresses, such as exposure to sub-minimal inhibitory concentrations of numerous antibiotics including beta-lactams [63] or ciprofloxacin (CIP) [64]. In *E. coli* [65] or *P. aeruginosa* [66], cell filamentation resulting from exposure to CIP sub-inhibitory concentrations was shown to be induced by the SOS response. Then, expression of *recA*, *lexA*, *uvrA*, *uvrC*, *lon* and *sulA*, previously shown to be involved in the SOS response [64,66], was measured in Pf4*-T condition (Figure S2). All genes were upregulated, except *lon*, in our conditions. Moreover, we measured a decrease in *ftsZ* transcription (Figure S2), which encodes a major cytoskeletal actor involved in cell division [67]. Altogether, these data suggest that Pf4* infection induces biofilm formation in *P. aeruginosa* by altering the architecture and cell morphology leading to the filamentation phenotype, which could be partly the result of SOS response induction.

### 3.4. Increased Matrix-Encoding Genes Expression and c-di-GMP Production in Response to Pf4 Variant Infection

**Figure 4:**
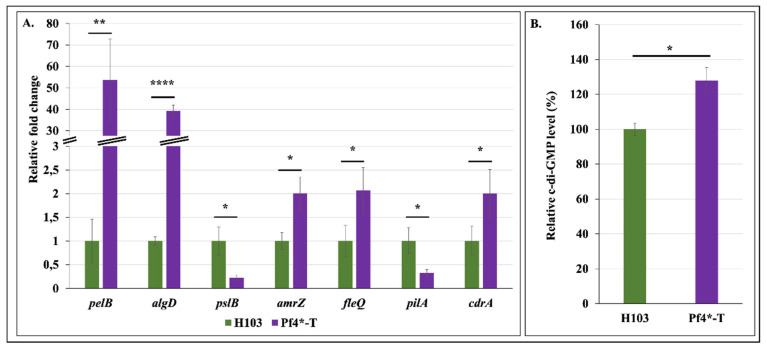
bis-(3′-5′)-cyclic dimeric guanosine monophosphate (c-di-GMP) quantification and matrix-encoding gene expression increase after Pf4 phage variant treatment. (**A**). Relative mRNA expression levels of *pelB*, *algD*, *pslB*, *amrZ*, *fleQ, pilA* and *cdrA* in H103 (green bars) and Pf4*-T (violet bars) conditions as determined by RT-qPCR experiments. (**B**). Relative c-di-GMP level measured by LC-MS/MS in H103 (green bar) and Pf4*-T conditions. Each experiment was assayed at least four times independently. Statistics were achieved by paired (two samples) two-tailed *t*-test. * *p* < 0.05; ** *p* < 0.01; **** *p* < 0.0001.

In the *P. aeruginosa* PAO1 biofilm matrix, the two exopolysaccharides (EPS), Pel and Psl, form a scaffold that structures the biofilm [68], while a third EPS, alginate, acts as a protective barrier [69]. Since it has been previously shown that biofilm architecture was closely related to the EPS produced by *P. aeruginosa* [70], we evaluated the expression of the three EPS-encoding operons by RT-qPCR. In response to Pf4* infection, expressions of *pelB* and *algD* were strongly increased by about 53.7 and 39.3-fold, respectively, while transcription of *pslB* was reduced by 4.5-fold (Figure 4A). It is recognized that AmrZ and FleQ are major regulators involved in EPS production [71](Maunders and Welch, 2017). It has been shown that the alginate and motility regulator AmrZ promotes *pelB* and *algD* transcription [72,73,74,75,76], while repressing *pslB* expression [77]. In addition, the master regulator of flagellar motility and exopolysaccharide production, FleQ, favors *pelB* expression [28,78,79]. Based on all these data, we speculated that AmrZ and FleQ expression and/or activity could be altered in the Pf4*-T condition. Accordingly, *amrZ* and *fleQ* transcriptions were increased by 2- and 2.1-fold, respectively, in response to Pf4* infection (Figure 4A). Thus, it is possible that the biofilm enhancement observed in the Pf4*-T condition was partly due to AmrZ transcriptional activity on *pelB*, *algD* and *pslB*, and of FleQ on *pel* operon. In addition, the AmrZ-repressed *pilA* gene [80] was strongly downregulated (Figure 4A), in line with the reduced twitching motility (as shown in Figure 2E), comforting a possible AmrZ activity. However, while significant, this two-fold increased expression of both *amrZ* and *fleQ* remains modest, suggesting that other mechanisms would be involved in the observed biofilm alterations in response to Pf4 treatment. FleQ is known to act on EPS production via its binding to the intracellular second messenger bis-(3′-5′)-cyclic dimeric guanosine monophosphate (c-di-GMP) [81]. High c-di-GMP levels are correlated to increased cell aggregation, surface attachment and biofilm formation in *P. aeruginosa* [82] (Romling and Galperin, 2017), via regulating expression of genes encoding enzymes of the EPS biosynthetic pathways [25,28,79,83]. We therefore quantified c-di-GMP level and showed that infection by Pf4* resulted in a significant increase of 27% of c-di-GMP level (Figure 4B). Interestingly, the *cdrA* gene, encoding a biofilm matrix protein [84], is regulated by c-di-GMP and its expression is therefore used as a c-di-GMP-level reporter [85]. As shown in Figure 4A, *cdrA* transcription was increased by two-fold in response to Pf4* infection, supporting our finding that c-di-GMP was overproduced in this condition. In addition, *cdrA* expression is regulated by AmrZ [74,75], and FleQ that binds to c-di-GMP to promote *pelA* and *cdrA* expression [79,84]. Altogether, these data show that *P. aeruginosa* H103 infection by Pf4 phage variant resulted in increased expression of genes involved in EPS production, at least via enhanced expression and activity of AmrZ and FleQ in the presence of high c-di-GMP level. Further assays would be required to establish a causal link between these two master regulators and the matrix EPS production as well as the biofilm alterations that were observed in response to Pf4* treatment.

### 3.5. Pf4 Phage Variant Infection Triggers a SigX-Mediated Cell Envelope Stress Response

**Figure 5:**
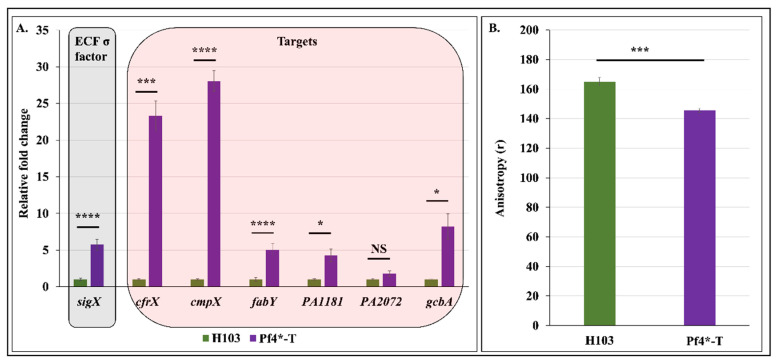
Pf4 phage variant infection triggers a strong SigX-mediated cell envelope stress response in H103 strain. (**A**). Relative mRNA expression levels of *sigX*, *cfrX*, *cmpX*, *fabY,* PA1181, PA2072 and *gcbA* in H103 (green bars) and Pf4*-T (violet bars) conditions as determined by RT-qPCR experiments. (**B**). Membrane fluidity assessment by fluorescence anisotropy using 1,6-diphenyl-1,3,5-hexatriene (DPH) probe in H103 (green bar) and Pf4*-T (violet bar) conditions. Each experiment was assayed at least four times independently. Statistics were achieved by paired (two samples) two-tailed *t*-test. ^NS^ *p* > 0.05; * *p* < 0.05; *** *p* < 0.001; **** *p* < 0.0001.

Since infection by Pf4 relies on attachment of phage particles on *P. aeruginosa* TFP and phage extrusion occurs across the cell envelope, we hypothesize that this phage–host interaction might trigger a cell envelope stress and thus a CESR. In *P. aeruginosa*, CESR relies mainly on the two ECF σ factors AlgU and SigX [29]. To assess the involvement of SigX in *P. aeruginosa* response to Pf4* infection, expression of *sigX* and some of its known targets (*cfrX*, *cmpX* and *fabY*) were evaluated by RT-qPCR. Remarkably, expression of *sigX* was strongly enhanced by 5.7-fold in response to Pf4* infection (Figure 5A). In addition, expression of the SigX targets *cfrX*, *cmpX* and *fabY* were increased by 23.3, 28.0 and 5.1-fold, respectively, in Pf4*-T condition (Figure 5A). Previous studies have reported inducing conditions of *sigX* expression [33,35,37,38,39,41,43]. For instance, the absence of the major porin OprF triggered increased *sigX* expression by about three-fold [33]. The exposure of *P. aeruginosa* to a cold-shock condition or to a valinomycin treatment resulted in increased *sigX* transcription by about two- and 7-fold, respectively [39]. Noticeably, the latter condition resulted in increased *sigX* expression at levels similar to the ones observed in response to Pf4* phage treatment. Since valinomycin as well as Pf4* treatment altered *P. aeruginosa* membrane fluidity, our data suggest that SigX is activated in response to membrane alterations. SigX is furthermore involved in c-di-GMP metabolism through PA1181, PA2072 and *gcbA* (PA4843) expression regulation [33,37,38,43]. As shown in Figure 5A, PA1181 and *gcbA* were upregulated in Pf4*-T condition, compared to H103. Since GcbA displays a diguanylate cyclase activity [74], while PA1181 contains both GGDEF and EAL motifs, our data suggest that these two proteins may be involved in the increased c-di-GMP level upon Pf4* infection. Interestingly, other growth conditions (e.g., high sucrose concentration and absence of the major porin OprF), in which SigX is active, were also shown to affect the expression of PA1181 and *gcbA* [38,65], suggesting that the CESR encountered by *P. aeruginosa* would be similar in these three conditions. In addition, SigX was previously shown to be involved in *pelB* but not *pslB* expression when *P. aeruginosa* was exposed to high sucrose concentration [38] or in a mutant lacking the major outer membrane protein OprF [33]. These two conditions lead to a cell wall stress, during which SigX is activated, suggesting a direct or indirect link between SigX activity and *pel* expression. Noticeably, *amrZ*, the product of which positively regulates the expression of *pel* but not *psl* in *P. aeruginosa* PAO1 [74], was recently shown to be part of the SigX regulon [39]. The *amrZ* expression was slightly increased in response to Pf4* infection, which correlated with SigX expression and activity. However, AmrZ was also described to be a repressor of *gcbA* transcription [74]. These data suggest that SigX could bypass AmrZ-dependent *gcbA* repression by a mechanism that needs to be unraveled by further studies.

### 3.6. Pf4 Phage Variant Infection Induces P. aeruginosa Membrane Fluidity Alteration

SigX has also been reported to be involved in the modulation of membrane fluidity through the regulation of fatty acid biosynthesis, which results in changes in the cell membrane phospholipid composition in order to maintain envelope integrity [37,41,42]. To evaluate *P. aeruginosa’s* membrane fluidity post Pf4* infection, fluorescence anisotropy (FA) measurements using a 1,6-diphenyl-1,3,5-hexatriene (DPH) fluorescent probe were performed. As shown in Figure 5B, FA values decreased when *P. aeruginosa* was infected with Pf4 phage variant, compared to H103, revealing increased membrane fluidity. Altogether, these data indicate that Pf4* infection increases membrane fluidity probably because of the strong activity of SigX. Interestingly, in *E. coli*, the ECF sigma factor RpoE (the AlgU homologue), the Cpx, Rcs and Bae signaling pathways, and the phage shock protein (Psp) response mediate CESR. Remarkably, the Psp response is activated in *E. coli* in response to infection by filamentous phages [86] and to treatment with the membrane stressors verapamil and dibucaine [87]. To date, no homologous Psp response was reported in *P. aeruginosa*. Our data might suggest that SigX would function in *P. aeruginosa* similar to the Psp response in *E. coli*.

### 3.7. Pf4 Phage Variant Infection Triggers an AlgU-Mediated Cell Envelope Stress Response

**Figure 6:**
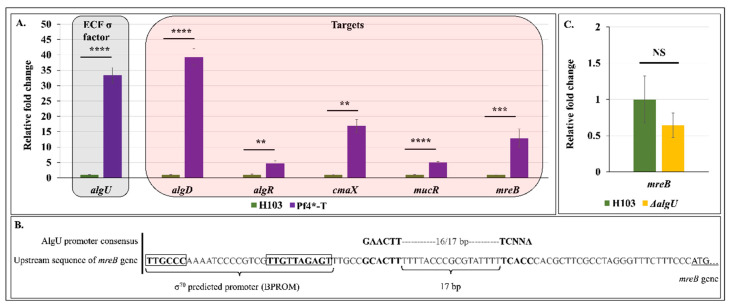
Pf4 phage variant infection leads to an AlgU-mediated cell envelope stress response in H103 strain. (**A**). Relative mRNA expression of *algU*, *algD, algR*, *cmaX, mucR* and *mreB* in H103 (green bars) and Pf4*-T (violet bars) conditions as determined by RT-qPCR experiments. (**B**). Sequence of the DNA region upstream of *mreB* showing a predicted AlgU-dependent promoter and σ^70^-dependant promoter. (**C**). Relative mRNA expression levels of *mreB* in *P. aeruginosa* H103 (green bar) and Δ*algU* (yellow bar) as determined by RT-qPCR experiments. Each experiment was assayed at least four times independently. Statistics were achieved by paired (two samples) two-tailed *t*-test. ^NS^ *p* > 0.05; ** *p* < 0.01; *** *p* < 0.001; **** *p* < 0.0001.

We next assayed expression and activity of the second major actor involved in *P. aeruginosa* CESR, the ECF σ factor AlgU. AlgU controls a large regulon of more than 300 genes, including *algU* itself, *algR*, *amrZ*, *cmaX* and the 12 alginate biosynthesis genes that *algD* is among [29,88]. Infection by Pf4* resulted in strong expression and activity of AlgU (see Figure 4A and Figure 6A), as was the case for SigX. The transcriptional regulator AlgR is involved in the expression of *algD* as well as *mucR,* which encodes for a diguanylate cyclase [89]. In the current study, we observed that *mucR* expression was increased by five-fold in response to Pf4* infection, suggesting that MucR could also contribute to the increased c-di-GMP level observed in *P. aeruginosa* after Pf4* infection.

Interestingly, the *mreB* gene, encoding an actin-like protein responsible for maintaining bacterial shape in many bacteria [90], was previously suggested to belong to AlgU regulon [88]. By visual inspection of the *mreB* upstream gene sequence, an AlgU-dependent promoter was found based on the previously described consensus sequence [34] (Figure 6B). Noticeably, *mreB* was overexpressed by 13-fold in response to Pf4* treatment (Figure 6A). However, *mreB* transcription level was slightly, but not significantly, decreased in an *algU* mutant, suggesting that AlgU was not involved or at least that other regulator(s) are involved in *mreB* expression in response to Pf4* treatment (Figure 6C). Moreover, a putative σ^70^-dependent promoter was found upstream of the AlgU promoter (Figure 6B), which could explain the basal expression of *mreB* in the *algU* mutant (Figure 6C). Since overexpression of MreB confers a filamentous phenotype, due to the inhibition of cell division [91], it is possible that MreB could contribute to the filamentous phenotype that we observed in Pf4*-T condition biofilms (Figure 3B). Further assays would be required to demonstrate the involvement of *mreB* in the cell elongation phenotype observed in response to Pf4* infection. This first set of data raised the question of the relationship between the membrane fluidity and the bacterial cytoskeleton. Intriguingly, in vivo observations using specific lipid-binding dyes showed that the membrane assembly of the actin-homologue MreB filaments with the membrane generates fluid lipid domains and promotes movement of membrane proteins and lipids [92], similar to actin in cortical cytoskeleton of eukaryotes [93]. In addition, MreB mis-localization induces formation of membrane invaginations enriched in high fluidity domains [94]. Based on these data, the increased production of MreB could generate a major stress leading to SigX hyperactivity and increased membrane fluidity. Taken together, our study reports, for the first time, the molecular relationships between Pf4* infection and the CESR in *P. aeruginosa*, involving both the two major ECF sigma factors in the bacterial response to such stress treatment.

### 3.8. Does the CESR Mediated by SigX and AlgU Rely on Pf4 Phage Variant Infection?

To further confirm the involvement of SigX and AlgU in *P. aeruginosa* H103 response to Pf4* infection, expression and activity of both SigX and AlgU were determined in the two ECF σ factors deletion mutants treated by Pf4*. As expected, no differential gene expression could be observed when comparing Pf4*-T to non-treated mutant strains (Supplementary Figure S3A), confirming that both SigX and AlgU are involved in the observed Pf4*-induced phenotypes. However, the signal resulting from Pf4* infection leading to their activity is still unknown. One possibility is that both mutants are altered in their TFP production and/or activity [43,95]. Since TFP are Pf4 receptors [12], their absence or inactivity could explain the absence of twitching motility and Pf4*-induced lysis plaques (Supplementary Figure S3B). To get further insights into the response to Pf4* filamentous phage infection, we infected *P. aeruginosa* H103 with LUZ19, a lytic TFP-mediated phage [45]. After 20 min of infection, corresponding to the very early entry into the lytic phase (before cell lysis, Supplementary Figure S4A), RNAs were extracted and expression of *sigX, algU* and some of their targets was investigated. Even if expression of *sigX* and its targets was slightly reduced when *P. aeruginosa* was infected with LUZ19, no significant difference of expression or activity was observed for both ECF σ factors (Supplementary Figure S4B), suggesting that the CESR mediated by SigX and AlgU may specifically rely on Pf4* filamentous phage infection. In addition, when the cells were first infected with Pf4 variant and further with LUZ19 phages, a clearly reduced lysis phenotype was observed (Supplementary Figure S4C), as expected since the two phages are competing for the same TFP receptors. Altogether, our data suggest that phage–host interaction via TFP is required but is not sufficient to generate a CESR. Otherwise, it remains plausible that the CESR was not activated after 20 min of infection. Further research is required to establish the specific molecular mechanisms of TFP activity inhibition after Pf4* phage binding and its relationship with the CESR.

## 4. Concluding Remarks

**Figure 7:**
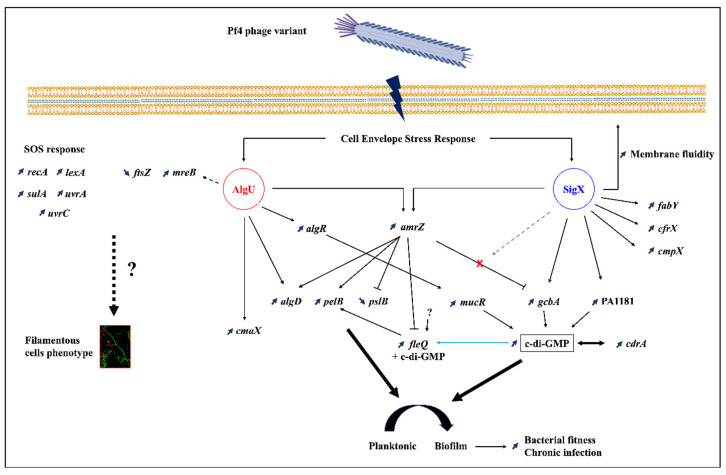
Pf4 phage variant infection altered *P. aeruginosa* H103 regulatory circuits and physiology. *P. aeruginosa* H103 infection by Pf4 phage variant leads to a SigX and AlgU-mediated cell envelope stress response. The activation of the SigX extracytoplasmic function sigma (ECF σ) factor triggers increased membrane fluidity and increased gene expression of *gcbA* and PA1181 diguanylate cyclases, most likely responsible of the c-di-GMP-increased concentration. The AlgU activation leads to increased *algR* expression, whose products are responsible for *mucR* increase, encoding another diguanylate cyclase. The ECF σ factors AlgU and SigX modulate *amrZ* expression, which in turn controls exopolysaccharide gene expression. The increased c-di-GMP concentration (according to *cdrA* gene expression) and exopolysaccharides lead to a switch from planktonic to sessile lifestyles. The increased of *mreB* expression and the induction of the SOS response could be responsible for the observed filamentation phenotype. Thin black full arrows or broken lines represent direct transcriptional regulation. Thin blue full arrow indicates a direct regulation, but not at the transcriptional level. Thin grey arrow represents the possible bypass of SigX in *gcbA* transcriptional AmrZ-dependent regulation. Thin dashed arrow represents potential regulation.

We show herein, for the first time, that Pf4 variant filamentous phage infection results in an extensive CESR, mediated by the two major ECF σ factors SigX and AlgU. Our data are summarized in Figure 7. Drastic phenotypes were observed in response to Pf4 variant infection at the cell-level, such as increased membrane fluidity related to SigX hyperactivity, and altered cell morphology. In addition to these important observed cellular phenotypes, Pf4 variant infection resulted also in increased biofilm formation and c-di-GMP production, at least partly via the activity of both AlgU and SigX. Since TFP serve as Pf4 receptors, their role in this major stress response would also deserve further studies, which would be deciphered at least partly by a global RNA-seq analysis. Finally, such Pf4 variant is an excellent tool to better understand the lysogenic bacteria response to phage infection. Additional studies on dH103Pf4^+^ strain would be of great interest to understand the relations, if they exist, between PA4722 mutation and Pf4 variants overproduction.

## Supplementary Materials

The following are available online at https://www.mdpi.com/2076-2607/8/11/1700/s1. Figure S1. Sequence of the whole Pf4* prophage variant from dH103Pf4^+^ strain (PA0715–PA0729). Figure S2. SOS response induction after Pf4 phage variant infection. Figure S3. Δ*sigX* and Δ*algU* mutants are resistant to Pf4 phage variant infection. Figure S4. Cell envelope stress response is not induced by LUZ19 phage TFP-mediated infection. Table S1. Strains used in this study. Table S2. Primers used in this study. Table S3. SNPs and deletion identified in PA0724 protein sequence from dH103Pf4^+^ strain.
