# Supplementary material for: Activation of the Cell Wall Stress Response in Pseudomonas aeruginosa Infected by a Pf4 Phage Variant
Source: Microorganisms. 2020 Oct 30;8(11):1700. doi: 10.3390/microorganisms8111700 (PMC7693463; doi:10.3390/microorganisms8111700)
Supplement: Supplementary file 1 [file microorganisms-08-01700-s001.zip › Supplementary Figure S1.pdf]

Legend:
Genes in blue
Intergenic region in black
Mutations highlighted in green

>PA0715-PA0729

ATGAAGAAGAGACCTTTAGAAGATCTTTTTTTTGCGATGTACAGAGGGAAGCAGAGTTTCGAACACTTT
GCCAATTGCTCTGTCGAAGGTTTATATGAGCCGGTGATGGTGAATGGACGACTCGTATATAAAACCGAT
AAGGATCTGCGTGCGTATCATCGGTTTCTAAATAAATTCTTGTTTGAAAGGCTTCCTGTTGTTGATGATG
TTGTGTTTTCTTATCGTAAAGGTGTTAACGCCGTAGATGCTGTCGAAAAGCATGCAGGTAGTAAGTTTTA
TTTTCAAACTGATTTAAGGGATTTCTTCAGTAGTCTAACTAAGTCTGATGTTAGAAGAACTCTGGAATGG
GCGAAAGATGTTTGTGTTATTTCAGACTTAGATGTTTGGGTTGAGAGAATTCTGAAGTTGGTTACTATTG
GTGATTCTTTGCCGATGGGGTTTTCGACTTCCCCTGCAATTAGCAATGCATGCTTATACTCTCTGGATGG
GATATTAAAGACGCATTGTTTAAGTAATGGTGTTATATATACTCGTTATGCAGATGATCTGATTTTTTCCT
CGTCGAACAAGCAGGCGCTTGATCCTTTGTTTGGGGTCGTGTGTCGTACCCTCCAATCTGAGTTTTCAGG
GCGGATGGGAATAAATTCACAGAAGACAAAGTACCTAAGAAAGGGGGGGAAAATTAAGCTGCTGGGGC
TTAATGTTCTTCCTAATGGAAGAGTTACTGTGGATATGCGGCATAAAAAAGAAATAGAGGTTCGTCTTTT
TTATTACGTAAAAGACAGAGCTAGGTTTTTGAAGATCATGGGCGAGGTGGATGATGAAAAAGCTATTAA
GAAATTCTCTGGTTTAATAAACTACGTAAATACTGTTGATCCGGCTTATCTTGATAAGCTTCGCAAGAAG
TATGGCTTAACGATTGTTGATATGTTTATTCATAAATCACCGAAGCTCTAGGTATGAATTTTCAGATAGT
AATAAATAATATCCAACATATTGGTTACGCTGAGCTTGATGTTGATTTAAATGCTAGTGGAATTATCTGT
ATAGTCGGGAAGAATGGAGTAGGAAAGACTACTCTAATTAAGGCTATTTTAAATTTAAAGTCTGCCGAC
ACATTTTCTAGAACCGCCTCGCCTGGTATTTTTAAGGCTAATAGCTCAATGCGCTGTACCTATGGAGAGA
ACTCATATGAATTCTCTTTTGATTCAGATATAAATGATCTAAATAGCCGATCCCCTATTCCTGAAGAGTT
AAAGTCTGTCATAGACGTTGAGTTACCTATGCCCTTTGGGCAGCGATTTAATAACTATCAAAATATTATG
AGCGCGGATATGGATATAAGAACCGCGTTGATATTGGGTGAGTACGAGGTACCCGTAGAGCTGATTAGC
TTCTTAAGTGATATTTATAAGACCAATAAATTTAATGGATTGGTAGAGGTCTCTGCAAAGGGGGGAAGT
TACTATTGTATCCCTTTAGATGATGATCGATATGTTCGCGAAGATCACCTCAGTTCTGGAGAATTTTTTTT
AATTAGTTTGTATAGGAAGATTAAGGGGCGTAGTAAGTTTATTGTCATTGATGAAATTGATATTTCTCTA
GATGCTGCGGCGCAGGCCCATTTAATTGGCTGGCTGCGGCGCTTTTGTACAACGGAACAGGTAAAGGTT
GTTTTTACTACCCACTCGCTAGCATTGATGAGGACATTAAAAGACGGTGAGTTGTTCTATATGGAAGAA
AGTGAAGGGAAAGTTAGTGTAATTCCTTCGTCATATAATTATATTAAAAGTGTTTTGTTTGGGTTTAAAG
GGTGGGATAGATATATTCTGACAGAGGATGCGATGTTGAAGTCTTTGTTGGAGTATGTTTTGGCTAACTA
TTGTTCTGGTCTTTTTTTTTCTTATCAGATTATCCATGTCGGTGGCGGGAGTAATGTGGTTGATCTGATGC
GCCAAAATTCCACCGAAGGGTTTTTCTCGACGCCCGAAAATGTCATTTCGGTGCTAGATGGGGACCAAG
CGGAGTATCGGTATGCTAGACGGCCCAATGTTTATTGCATTCCATTCTTGAGCGTTGAGAAAGCAATTTT
TGCAGAGTACAAGGCCGGAAGATTTTTGCCGGAATTGGCGCCTGATCCTCGATTTGATACGCCCAAGAA
TTTTTGTTCGGCAATAGGTCGCGACCGACATGCAACTGAAGCGGATATAATTAAGTATCTTTGTGATAGT
TATCCAACGGAAGTGCGTAGATTTGCGCAAACCCTTAGTGACTTCCTTAGTTGATGGCATATATTCTCGG
GGCAGCATGTGTTTGTCGCAGCAATCGAACCCAATTTTCTACTTACGCCTAGAATGCGGAACTAATTTCT
CAGTGCTGACCTGTGAGAGGTGATTGAGGGGTAAGGCGGGAGTCATCGCAATTCTCATGTTTTCTCGGA
GTTTTTGGGGGGGGGCTTGACCAAGGAAGTTAAGGACAATCGTCGTCTATGAGATTACCGTATCCTTGTC
AGCTTCATTTCTTGCCTTCCATCCGTAGGGCATACCATCGCAATGCTACTTTTCTAGTGATCGCTATCCCG
CGTTTTGATTGGACAAGTTTCGATTGGCTTCGTCATAGTCGGGGCTAGTTTGGCCGACTTCTGGGGCGAT
GGAACCGCTTGCAATCCATAATGCGTAATTAGGGAAAATTTTTACTAGGACGTCGATTTCTTCCGTGCTC
ACGCGAATCGCGCCTTTGCTGACGCTTTTCCATCTCTCGTAATCGCCCCCGTGAAGGCTGACCTTTTTGG
GGCCGATTTTCTTGATTAATAGTCTTGCTCTATCGGCTGACGTGCTCATATAGAAATTTATTCCGGGGAA
ATAGTTGCCCTGACGTGGCTTTGGGGAAATAATTTCTCCAGGGTAATTATTTCTCTAGCTGCGTATGGCT
AATGCCACGAATAGTGACGGAATGAGCATGGAACTGGAAGAGCTTAGCCCCAGTGCCCTGATAGGGCC
GCAACAGGATGTGGAGTCCATCGAACGGTGGGCGGAGCGCAACGGCATCAGCTACGGCACCGCTCGCG
CCTGGGTTTACCGGGGTGTGCTGCCGTCGGTGAAGCTGGGGAAGCTGCGCATGGTGAATAGCGCGCTGC
TGCGTACCTGGCTGTTGGAACAGGAATGGAGTGCCTGATATGCGCTACCTCGTAGAGATTTGCACCTTCC
ACGGCCCGACACGGCAACGTCGCTGGCATCGCGTCCATCAGGGCGGTTCCCGCGTGGAATGCCAACGCT
GGGTCGAAGAGTTGGTGGCTGTCTTCCCGACTGAAGAAGAAGCTCGCCGCTCCTTCGGCCTGACCCGCG
AACGCGCTCGGCAGGTGTACCGCATCCGTGGGGTGAGGGCATGAACCATGGCCGCCAGTCCCTACTACC

TACGCCAAACCCACGCCCCGGACTGCGCCTGCTCTGTGTGCTGGTCCGCAAGGCAGGTCATCCCATTGC
ACAGCCCGTCGCCGTGTCCAGACTGTCGGCCCCCTGGGCTGCCCTATCTGGAAGGTGGCCGCTGGCTCTG
CCGTCCCCGTTCCTTCTGCGCGAAACACGACCCGTCCCGGCGTCCGCCGAAGTACTGGCACGTTGTGTAC
GACAGCGGGAAGCCCACGCCCTTTGTGCCCGTGCGCGAAGCATTCCAACTGGAGGGCTGAACCATGCTT
GCTAAGACCCTGAAAGCGCTGCTCCTGCTCTGCCTGATCCAGGCCGCCCGCACCGTGGCCGATCCGGTC
AAGGGCCGCGCTCCCGGCTCGTCGGAACAGCCTCACCGTTCCGGCGAACGGAAGCACGGGCGGAGCGC
ACCCTTGAACGCCTCCCCCCTGAAACAGCCTCCGCTGGGGAGTGTGGGGCAGCTTCTCCGCCCCGCGCTC
CCGAGCCCTCGGCGGCAAGAGCGGGATGACAAGGGCAGAGCCCTTGGTGTTGCTCTGCGGGTTCCAAGG
GGAAGCGTTCCCCTTGGCCGTCGGAGACGACGTTGCGATAGGGATCGTTCCCCGAATGGGCCGAGACGA
ACACCCGTGGTTGGCTTGGTTCGCTAGCGAATAGAGCCCGGCCCGAAGGGATCGCCCGACAAATCACTT
TCACCCAACACCGCTGAATGAAGGCGAAACAGCCGAATTTGCAGCAGCGGGACAACTCACGCCGAAAA
AGGCGAATTGAAGGAGAAACACCGATGAACATGTTTGCAACCCAAGGCGGCGTCGTCGAACTGTGGGT
CACCAAGACCGACACCTATACCTCGACCAAGACCGGGGAAATCTACGCCTCGGTCCAATCCATCGCCCC
GATCCCGGAAGGTGCCCGTGGCAACGCCAAGGGATTCGAGATCAGCGAATACAACATCGAGCCGACCC
TGCTGGACGCCATCGTCTTCGAAGGCCAGCCGGTGCTCTGCAAGTTCGCCAGCGTGGTCCGTCCGACCC
AAGACCGTTTCGGCCGGATCACCAATACCCAGGTCCTAGTGGATCTGCTGGCCGTGGGCGGCAAGCCGA
TGGCGCCGACCGCCCAAGCCCCGGCCCGCCCGCAAGTGCAGGCCCAAGCCCCGCGCCCGGCCCAGCAG
CCGCAGGGCCAGGACAAACAAGACAAGACCCCGGACGCCAAGGCGTAAGCCCTAGGAGGCCGCGATGC
TCCGCTATCTCTCGCTGTTCGCGGTAGGTCTGGCCACCGGCTACGCCTGGGGCTGGATCGACGGCCTAGC
GGCCTCCCTGGCTGTTTGAGGACTGATCGCTATGTCAGGCGTTGTCGCTGTGCAGGTGTGTACCGCGTGG
ACCTCGACCCCCGAGGGCTTCATGGCGTGTCGCGAACTCGCATGGCAACAGGCCTACCTGATTCCGCCA
GAGGCCGCTGGATACGTGGACATCCTGGTCAACGGTGGTTTCTCCCCGGAAGCCTTCGGCATCGGTGCC
GCTGGCGTCCTGGGATCGTTCGTGACGGGGCTTTTGATTGGCTGGGTCGCGTCACTTCTTCGTAAAGCCA
AGTAGAGAGGAAACACCATGAAAGCAATGAAGCAACGCATCGCCAAGTTCAGCCCGGTCGCCTCGTTC
CGCAACCTGTGCATCGCCGGTTCCGTCACTGCCGCGACTTCGCTGCCGGCCTTCGCCGGGGTGATCGACA
CCAGCGCGGTGGAATCGGCGATCACCGATGGGCAGGGCGATATGAAGGCCATTGGCGGCTACATCGTC
GGCGCCCTGGTGATCCTGGCCGTCGCCGGCCTGATCTACAGCATGTTGCGCAAGGCGTAACGGGTGCTC
TGGTCGGTGTGGTTGGGGGCGTTCTTCGCCGGCGCCTTCATCACCGGGTACCGGACCGGCGAATTCTTCT
AACCGAACAGACCGAGGCGGAAGCCCCCTCCGGAGTTTCCGGCAGGGGGCTTTTTTGTGTGGGGTCTTA
CGATGAAGTTTGCGAGCCTGATTCTGATGCTTCTCTTTGCCACGGTGGCGAGGGCTGAGGATTACTACTG
GAAAATTCAGTCACTGCCTGAACGCTTTTCTTCGCCCTCGGCAGCTTGCGCGGCGTGGGCCAAAGCCAC
GGGACGCCCTGGGGAGTTCACCTTCACCGGGTCTATGAAAGCCCGTGACCAGACCTCGTTTTGGTGCGA
GTTCACGAACAACGAAACCGGCAAGACTGCTGCCGGGTATGGTCCTGCCGGACGCTATGGCGATAGCTG
TCCCGAGGGGACGGAATACGATAAGGCGACCGGGGTTTGTAAGTCGCCTCCGCAAGAATGCAAGGAAG
GCGAACTGTTCCCGGCCAAAGGCCCGGACTCGCCCGTGGTTACCTCGGGAGGCCGTAACTATGTCGGTG
ACGGCGGCGCCCCGACCGCCTGCTATCAAAGCTGTGAGTATGGCGGCAATCCCAGCCCGGCCAGTTGCT
ATTTGGTCAAAGGCTCCACCACGACCGGCTTCTGCAATTACATCCTCAAGGGCACCGGCCAAAGCTGCG
GTGCCGACTCCTACACCTTCTCCCAGACCGGCGATTCGCTGAACCCGCCCGACACTCCGAACACCGATCC
TTCCGACCCGAACGACCCCGGCTGCCCGCCCGGCTGGTCGTGGTCGGGAACTACCTGCGTCAAGGCCCC
GACCGATCCCACGGATCCAACCGACCCGACCACGCCGGGCGGTGATTGGCGACGGCGATGGCAATGGCG
GTGGAAACAACAACGGCGGCGGCAATGACGGCGGCACCGGCAATGGTG---
ACGGCAGCGGGGGAGGGGACGGCAACGGCGGGGGCGATGGTAGCGGCGACGGTGACGGCAGCGGCAC
GGGCGGCGATGGCAATGGCACCTGCGACCCGGCGAAAGAGAACTGCTCCACCGGCCCAGAAGGCCCTG
GCGGCGAACTCAAGGAGCCCACGCCCGGCACTTGGGATGACGCCATCGCCACCTGGGAAAAGAAGGTC
GAGGAAGCCAAGCAAGAACTCAAGACCAAGGTGAAGGCCAACGTCGACCAGATGAAGGGTGCCTTCGA
CCTCAACCTGGCGGAAGGCGGCGGGCAACTGCCCTGCGAGTCCATGACCATTTGGGGCAAGTCCTACTC
CCTCTGTATCTCCGACTACGCCGGCCAACTCTCCAGCCTGCGCGTGGCGCTGCTGCTAATGGCCGCGCTG
ATCGCTTGCCCTCATTCTGCTGAAGGACTGACCCTATGGAATGGCTCTCCGGTTTTCTCGATCAGATCATC
GCCTTCTTCCAGTGGATCTGGGATTTCTTCGCCCAAGGCATCTATGACTTCGTGCGCGACGGCCTGGTGG
TCGCCACCAAGGCGTCGATGTACGCCGCGCTCCAGACCCTGATCCTGCTGATCGATGTCAGCTACACCG
CCGCCCGCGAACTGATCGACAGTCTTGGCGTGCCGCAGATGATCCGCAGCATGTATGCCGCGCTGCCGG
GGCCGATTGCGGCGGGGCTGGCCTTCTTCGGCGTGCCGCAGGCGCTGAACATCATCATGGTCGCGGCGG
CGACGCGCTTCTGCATGCGCTTCGTGCCGTTCATTGGGAGGTGATCCGTGTCGATCAAGATCCACCACGG
CCCCAATGGCTCCTACAAGACCTCCGGCGCGATCCAGGATGACGCCGTGCCCGCGCTGAAAGACGGGCG
GGTGATCATCACCAACGTGCGCGGCTTCACCCTGGAGCGGGCCTATCAGGTCTTTCCGGACCTGCCCAA
CACGGCGGAAATCATCAACCTCGATCTGGAGTCGCTGGAAGACCTCGAAAAGATGCGCACGTGGTTTCA

GTGGGCGCCCCGCGGTGCCTTCCTGATCTTCGACGAAACCCAACTGCTGTTTCCCAAATCCTGGCGGGAA
AAAGACCTCGAGCGCTTCGACTACCCCGGTGGACCGGAAGCGGCCCACGCGGCCGACCGCCCCATGGG
CTGGCTCGACGCCTGGACCCGGCATCGGCATTTCAACTGGGACATTGTCCTCACCACGCCGAACATCTCC
TACATCCGCGACGATATCCGCATGACCTGCGAGATGGCCTACAAGCATTCCAACCTCGCGGTGATCGGC
ATCCCTGGCCGCTACAAGGAGGCCCAGCATGACGCCCAACTCAACCGTCCGCCCGCCGATGGCACCATC
ATCGAGTACAAGCGGATCCGAAAGCAGACCTTCGCCCTCTACCAGTCCACGGCCACCGGCAAGACCCAG
GACACCAAGGCGGGCAAGAGCCTCTTCCGGTCGCCTAAGCTGGTTCTTCTACTGGCATTGCTGGCCGGC
ACTATTGGCTTTGTCTGGTATATGGGGCCTCTGCGCACGATTGGCGCTCCGGCTGCTGCGACACCTGCCG
ACGCTCCTGGCGACCCTGCTCAAGCCCCTGCTGCGCCCGCTGCTGTGGCTGCTCCAACGCGTCCTGCTGC
GAATAGCTTTCTTCCTCCTGGGCTTGTACCTGATGGGCCTGCTGCTGCGCCTGTTGATCTGAACGCCCAT
CCCTTCGCCGATCGGCGGATCTCCATCCTTGCCCACGCCTACCGCAAGTCGCGGGGCGACATTTACATGT
TCGCCCTGGACGATCCCACGGGCCGGCGCCTGGAACTCACCAGTTGGCAACTGATCGGCTCCGGCTACC
GGGTAACGCCCAAGGGCGAGTGCGTCGTAGAGCTTCGCTATGAGGAGTGGAAACAGACCGTCACCTGT
ACCGGGAGGCAGCCCGGCGCGGTGGCCAGCATCGTTCCGGCAGCGCCTGTTGCCGCCTCCGCAGACGCA
CCGGCCAGGGGGCAGTCGCCGCTGACCATCGTCCCCGATTCCGAATACGCCTCGCGGCCCTGGAGGCAG
AAATGATCGATTGGGAATTCCTCGCCCCGGTGGCGATGGGCTGGGCGCTGCATCACTGGTGGACGGTGA
TGACGGCGCTAGCGGCGGTAGGGGTGCCGCCATGAGGGGCGGGCCGCGCCGCCGGCCGGGAGCGCAAG
GCATGAGCGATAGGCCGAAGGCGCGGCCGACGCCCCTGTAACACGTCAGATAAGCCACCTATTGCGGTT
TCAATTCGTACCAATTTGGATCGTTAAAGATGAAGAAAATCAGCCATCAAATTCGCGTCAGTATCGAGT
CGGACGGTCAGGTCTTGGAAAGCCCGAAAGGGCGGTTGTTCTTCGACGACACCACGGCTCAATTCACCG
ACCTGTCAGGCGTGCGCATTCTGCGGTGCGGCGTGGATACGGTGCGGCAGTTGTACAACGGCAAACTCC
GGCCGGAAGTCATGGCGCTGTTTGACCTCTCGGTGGATGTGGTCGAGTTCGCCGGCTACGAGTGGTCCA
AGGGCCGCATCGGTCGCGACTCCGGCTATCAGTACCGCCTGCAGAACGCTGAAATGGGTCTGATCCTGC
TAATCAAGAATCACAACATCAAGGTCGACACCATTGGCTCGCACCTCAAGATCGAGGTATCGCCTCACG
CCCTCGATGGCGCCGATCCGCGCATCCTCCAGGGCGTGCTGGATGATTTGGCCGCTGCCGTGCTGAGTCA
CTGCGAAACCAACCAAGCCGCTGTGCATATCGCCTTGGACGTGCAGGGCTGGAAACCGCCTCGCGATCT
GGTGGACCGCATGCATTGTCGCTCGCGTCGGGTGCGACAAATCAGTGGGATCGAGCGGATCGAATTCGA
CGGCAACGCCTCGGTCTACGGGCGTGGCGAGACGTACATGTTCGGCTCGGCCAACGGCCTGCAACTGTC
GATCTATAACAAGACCCTCCAGGCTCGGGCCACCGACAAGCTCGACTATTGGGAAAGCGTGTGGGCGAC
CCTGAACGGGGATCCGTTCGGCGATGGCGACCCGGCCTATAACCCCCTGGAAACGGTCTGGCGGCTCGA
ATTCCGCTTCCATCACTCCATCGTCCAGCAGTTCTCCGAAGGCTCGCGCATGGCTTCGGGAGAGGTCATT
GGCTGCCGCACCTATGAGGGGCTTTGCCCGCACCTGCAAGGACTGTGGAACTACGCCTGCGAAAGCTTC
AAGCTGCTGAGCCGGACGGCGGTCTACGATCCGTTCTGGAGCCTGATCAGCCAGGACGCCCGCGTCCAG
GTCGAGTGCGATCCGCTGATCGAGCGCACCGAGTATCGGCGCTATTACAAGACCGCCAAGGGCTTTAGC
GGGCGTAACTGCGAGATGTTTCTCGGCCAGTTCGTGAGCCTGATCGCGCGGGAGCGTGTCCCGGCAAAA
AAGGCTATTGAGTCCGCCCGTAAACTGGAGTTCTGGCACGTTATCGAAGACCACTATCTCGCCAAGGGT
TGGACTCGTCGCGATCTGGAAAGGCACATACACAAGCTGATGTGTGATCGGTATCTGCGGCGGGGGTAT
GCCGTCTAATGTCGATCACCAAGCTCCCCGATGGCCGTTGGTTCGTCGATGTAGAACCGATCAAGGGCA
AGCGCTTTCGCAAGCGGTTCAAGACCAAGATGGAGGCGCAGCAATTCGAGGCCACCGCGCGTCAGAAG
TGTGCGGAAAACCCCTGCTGGACGCTCAGGCCGAAGGACCGTCGGCGTCTCTCGGAGTTGGTCGAACTC
TGGTATGAACTGCACGGCCAGACCTTGAGCAACGGGCATCGTTGCGTGGCGATTCTGCGGTTGGTGGCA
AAGGACCTGGGCGACCCGGTCGCTGTCTCCCTGGAGCCTGCGAAAGTGGCTCGGTTGCGTAGCCGACAG
ATAGCCAATGGCATGTCGGGCAAGACCGCGAACAACCGTCTTGGCTACCTCAAGTCCATGTACAACGAA
TTGCGCCAACTCGGCGTCATTGACTATGAGAATCCGGTAGGGCGCATGCGGCCGCTCAAGCTTCAGGAA
AGACCGTTGTCGTACCTGACCAAGCATCAGGTGTCCGAACTGCTTACGGCCCTGGATGCGCGCACCACG
TCGCCACATCCGAAGATGGTCGCTCGTATCTGCCTCGCGACAGGGGCTCGATGGGGTGAGGCTCAGGCG
CTGACGCCGGAACGTCTGAAAGGTAATACGGTGATCTTTGCCAACACTAAGTCCAAGCGTGTGCGCTCG
GTGCCGATCTCGGAAGAATTGGGCGCCGACCTTCGCCGGCATTGGCAGACCCACGGGCCGTTCACGAAC
TGCCTTGGCGTGTTCCGCCTGGTGCTGCTGTCGACCTCGATCAAGCTGCCGAAGGGGCAGGCCAGCCAC
GTACTGCGCCACACGTTCGCCAGTCACTTCATCATGAACGGCGGGCACATCGTGACCCTACAGCACATC
CTGGGGCACGCCTCGTTGTCGATGACGATGCGATATGCGCACCTCTCCCAAGACCACCTATCTGAGGCT
GTTCGATTCAACCCGCTCATAGGTTGAAGGCTGCGGGGGTCGACAGAGGGAAAGAAAAATAGACTTGA
GGTGGTTCAAATTCGGTCTGAATTCGGATTATGATGTTGGAGCCGACGGTAGACAGACTGCCGACGCGC
GAATCCCACTCGTCGCCTGGATATGGAGCGTGGTGGAGTTCGAACACCGTAGAACCTGAGTTCCAGGCC
TTAAGTGTTCCCACAGCAATGGAGGTACCGGCTCATGCGAGTCGAGACAATTAGTTATTTGAAACGTCA
TGCGGCTGACCTGGATTTATCCGAGCCAATGGTCGTCACGCAGAACGGTGTTCCTGCCTATGTGGTTGAG

TCATATGCTGAGCGGAAGCAGCGCGATGAAGCAATTGCGCTGGTGAAGTTGCTTGCGATTGGCTCCCGC
CAGTACGCAGAAGGCAAGCATCGCTCTGTTGATGATTTGAAAGCTCGCCTTTCCAGGAGGTTCGCTCAG
CCAGAATAAGGAGGTTTAATGTCCCCGGTCGTCATTCGTTTTACTGATACCGCAGAGCAAAGCATCGAA
GACCAAGTCCACCACTTGGCTCCATTCCAAGGTGAACAGGCTGCACTCCAGTCAGTACTGAGCCTTTTGG
ATGAGATTGAAGAGAAGATTTCACTTGCACCTAAAGGTTACCCAGTCAGCCAGCAGGCGAGTCTTCTGG
GGGTGCTGAGCTATCGCGAGCTTAATACCGGCCCCTATCGTGTTTTTTACGAATTCCACGAAGAGCAAG
GCGAGGTGGCAGTGATCTTGGTTTTGCGACAGAAGCAGAGCGTTGAGCAGCAATTGATCCGCTACTGCT
TGGTGGGGCCAATCGAGTGA

Supplementary Figure S1. Sequence of the whole Pf4* prophage variant from dH103Pf4^{+} strain (PA0715-PA0729). Genes were represented in blue and intergenic regions in black. Mutations in Pf4* genome compared to the Pf4 prophage wild-type genome from PAO1 reference strain (www.pseudomonas.com, Winsor et al., 2016) were highlighted in green.
