# Supplementary material for: Activation of the Cell Wall Stress Response in Pseudomonas aeruginosa Infected by a Pf4 Phage Variant
Source: Microorganisms. 2020 Oct 30;8(11):1700. doi: 10.3390/microorganisms8111700 (PMC7693463; doi:10.3390/microorganisms8111700)
Supplement: Supplementary file 1 [file microorganisms-08-01700-s001.zip › Supplementary Figure S2.pdf]

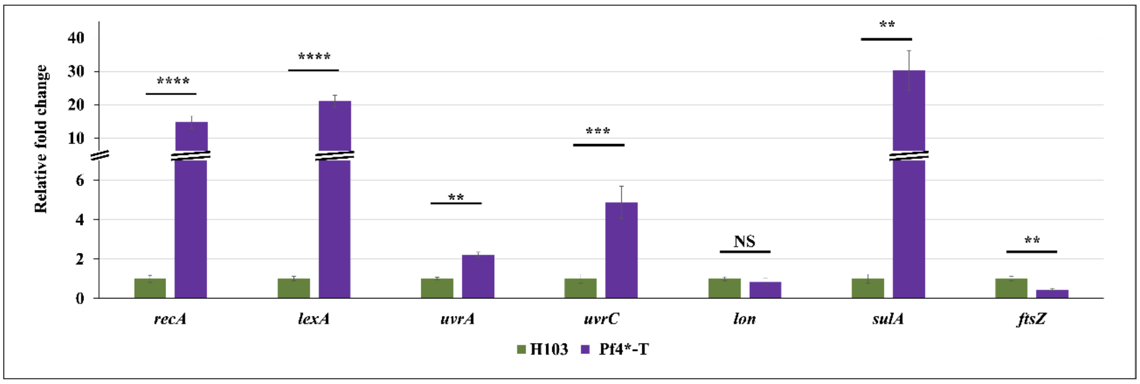

Supplementary Figure S2. SOS response induction after Pf4 phage variant infection. Relative mRNA expression levels of *recA*, *lexA*, *uvrA*, *uvrC*, *lon*, *sulA* and *ftsZ* in H103 (green bars) and Pf4*-T (violet bars) conditions ± SEM as determined by RT-qPCR experiments. Each experiment was assayed at least four times independently. Statistics were achieved by paired (two samples) two-tailed *t*-test. ^{NS} $p > 0.05$; ** $p < 0.01$; *** $p < 0.001$; **** $p < 0.0001$.
