# Supplementary material for: Activation of the Cell Wall Stress Response in Pseudomonas aeruginosa Infected by a Pf4 Phage Variant
Source: Microorganisms. 2020 Oct 30;8(11):1700. doi: 10.3390/microorganisms8111700 (PMC7693463; doi:10.3390/microorganisms8111700)
Supplement: Supplementary file 1 [file microorganisms-08-01700-s001.zip › Supplementary Figure S3.pdf]

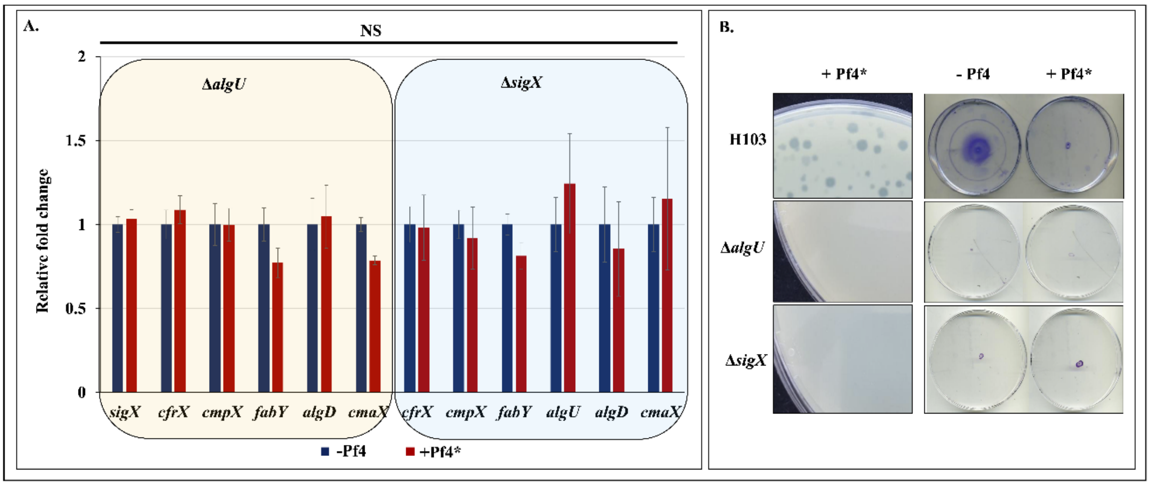

Supplementary Figure S3. Δ*sigX* and Δ*algU* mutants are resistant to Pf4 phage variant infection. Relative mRNA expression levels of *sigX*, *cfrX*, *cmpX*, *fabY*, *algU*, *algD* and *cmaX* in Δ*algU* and Δ*sigX* mutants treated by Pf4 phage variant (red bars) or not (blue bars) as determined by RT-qPCR experiments. B. Plaque lysis assay and twitching motility in *P. aeruginosa* H103, Δ*algU* and Δ*sigX* mutant strains treated or not by supernatant of dH103Pf4^{+} containing Pf4* phages. Each experiment was assayed at least four times independently. Statistics were achieved by paired (two samples) two-tailed *t*-test. ^{NS} $p > 0.05$.
