# Supplementary material for: Activation of the Cell Wall Stress Response in Pseudomonas aeruginosa Infected by a Pf4 Phage Variant
Source: Microorganisms. 2020 Oct 30;8(11):1700. doi: 10.3390/microorganisms8111700 (PMC7693463; doi:10.3390/microorganisms8111700)
Supplement: Supplementary file 1 [file microorganisms-08-01700-s001.zip › Supplementary Figure S4.pdf]

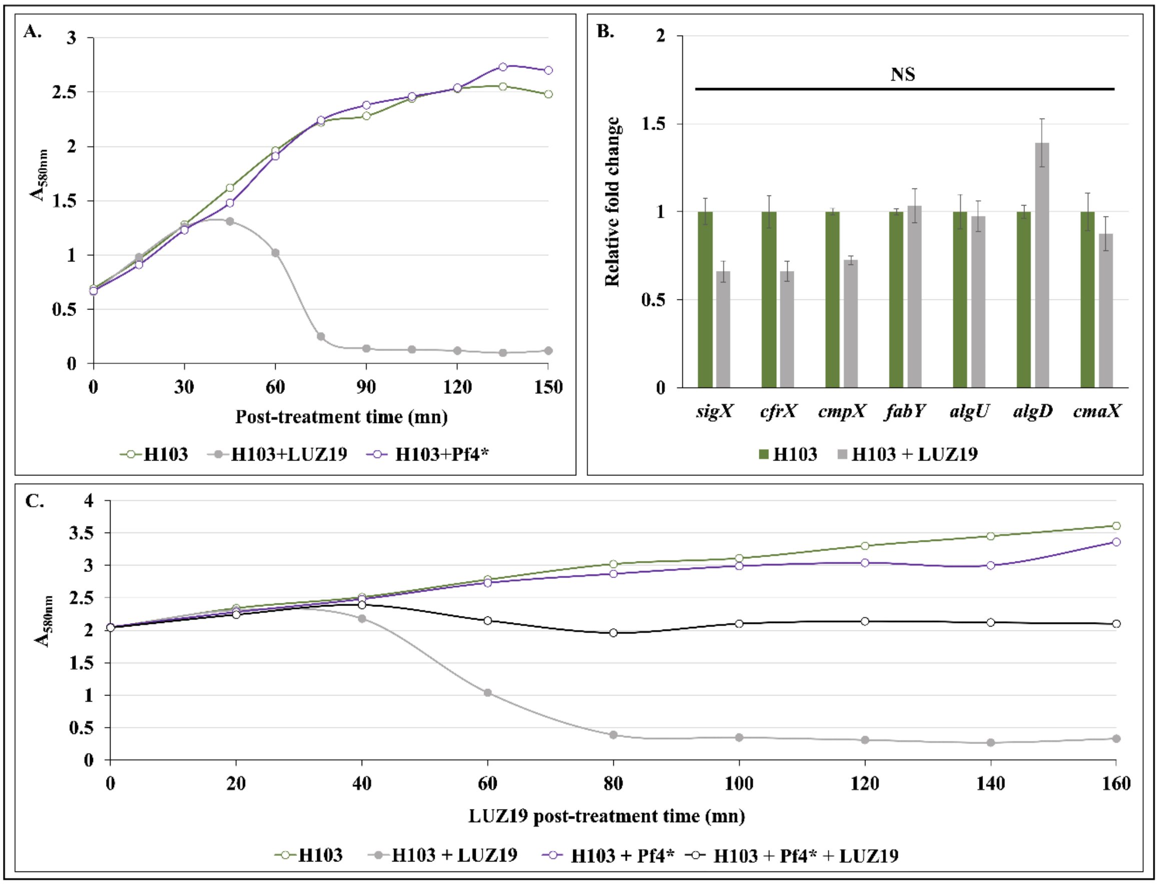

Supplementary Figure S4. Cell envelope stress response is not induced by LUZ19 phage TFP-mediated infection. A. Growth curves of H103 (green curve) strain treated by Pf4* (violet curve) and LUZ19 (grey curve) phages. B. Relative mRNA expression levels of *sigX*, *cfrX*, *cmpX*, *fabY*, *algU*, *algD* and *cmaX* genes in *P. aeruginosa* H103 treated or not by LUZ19 phage as determined by RT-qPCR experiments. C. Growth curves of H103 (green curve), H103 treated by Pf4* (violet curve) or LUZ19 (grey curve) phages, and H103 treated first by Pf4* phage followed by LUZ19 phage (black curve). Each experiment was assayed at least four times independently. Statistics were achieved by paired (two samples) two-tailed *t*-test. ^{NS} $p > 0.05$.
