# Supplementary material for: Activation of the Cell Wall Stress Response in Pseudomonas aeruginosa Infected by a Pf4 Phage Variant
Source: Microorganisms. 2020 Oct 30;8(11):1700. doi: 10.3390/microorganisms8111700 (PMC7693463; doi:10.3390/microorganisms8111700)
Supplement: Supplementary file 1 [file microorganisms-08-01700-s001.zip › Supplementary tables.pdf]

Table S1. Strains used in this study.

Strains	Relevant characteristics	Sources
Pseudomonas aeruginosa		
H103	Wild-type strain; prototroph derivative of PAO1	Hancock and Carey, 1979
Δ*sigX*	H103 *sigX* deletion mutant	Bouffartigues et al., 2012
Δ*algU*	H103 *algU* deletion mutant	Bouffartigues et al., 2020
PAK	Wild-type strain	Laboratory of A. Filloux
dH103	H103 derivative strain with chromosomic insertion of Mini-CTX-Lux vector	This work
dH103Pf4^{+}	dH103 overproducing Pf4 variant phage (Pf4*)	This work

Table S2. Primers used in this study.

PA number	Gene name	Primer name	Sequence (5' – 3')
**PA0668.1**	16SrRNA	16S-F	AACCTGGGAACTGCATCCAA
		16S-R	CTTCGCCACTGGTGTTCCTT
**PA0717**	PA0717	PA0717-F	CGTAGAGATTTGCACCTTCCA
		PA0717-R	ACAGCCACCAACTCTTCGAC
**PA0762**	*algU*	algU-F	CACGCCCTGAAGGACATC
		algU-R	CAAATCCTCGGGCAACTG
**PA1097**	*fleQ*	fleQ-F	CGCGAAGACCTCTACTACCG
		fleQ-R	CGAGATCAGTTCGTTGAGCA
**PA1181**	PA1181	PA1181-F	CCAGATGGAGAAGCGCTACCT
		PA1181-R	CGCTTGCGACTGTCGATATC
**PA1727**	*mucR*	mucR-F	CCACGAGCTGATGATCAATG
		mucR-R	TCGAACTCTCGAAGAAGCAG
**PA1773**	*cmaX*	cmaX-F	AAAGGGCCGAAGACAGCAT
		cmaX-R	CGCGATCGGTCAGGTAGTG
**PA1774**	*cfrX*	cfrX-F	CTGCGGGACCTGCTCAAG
		cfrX-R	GCCGACCTGGCGATTG
**PA1775**	*cmpX*	cmpX-F	TATCTGGACCCAGAGCTTGC
		cmpX-R	GAAGCCGAGCAGAACGAC
**PA1776**	*sigX*	sigX-F	AATTGATGCGGCGTTACCA
		sigX-R	CCAGGTAGCGGGCACAGA
**PA1776**	*sigX*	mut-sigX-F	TTGGCTCGTCCATGTCAATC
		mut-sigX-R	CATGATATCCGCTATCTCCTGAAA
**PA1803**	*lon*	lon-F	CAGTTGGGCAAGAAAGTCC
		lon-R	TGCTTCTGCTCGATCTTCAG
**PA2072**	PA2072	PA2072-F	CCAGGCATCAGGACGACAT
		PA2072-R	CGATTCTGCAGCGCCTTT
**PA2232**	*pslB*	pslB-F	ACACCAACGAATCCACCTTCA
		pslB-R	CGCTCTGTACCTCGATCATCAC
**PA2585**	*uvrC*	uvrC-F	AGGACAGCTATTTCCGCAAC
		uvrC-R	TTCCTTCGAGGAACATCACC

**PA3007**	*lexA*	lexA-F	AAGCCGAGATCCTCTCCTTC
		lexA-R	GACTTGAAGCCGAGTTCCTG
**PA3008**	*sulA*	sulA-F	GTGGTCAGTTGGCTGGAAC
		sulA-R	TATTCAGGCTCTGTGCTTGG
**PA3063**	*pelB*	pelB-F	CGGCTACGTGCAGCGTTAT
		pelB-R	CACTGCATGCGTTCCTTGAC
**PA3385**	*amrZ*	amrZ-F	CGTGAGCAGATCGCAGAA
		amrZ-R	GCTCGTGCAGGCTGAGTT
**PA3540**	*algD*	algD-F	GATGCTCGGTTCGTTGATG
		algD-R	TCGTGGCTGGTGATGAGAT
**PA3617**	*recA*	recA-F	GTAACGCACTGAAGTTCTACGC
		recA-R	TGTTCTTCACCACCTTGACG
**PA4234**	*uvrA*	uvrA-F	ATCGTGATCACCGGTCTTTC
		uvrA-R	TCCATCATCGACAGGAACTG
**PA4407**	*ftsZ*	ftsZ-F	CATGAGCGAAATGGGCATGG
		ftsZ-R	AGGTTGACGTCTTCCAGCAG
**PA4481**	*mreB*	mreB-F	CAAGGTCCACGAGAACAGCT
		mreB-R	TTCGATCAGGAACACCTCGC
**PA4625**	*cdrA*	cdrA-F	GACGCCTACGTCAACAGTCA
		cdrA-R	GTTACCGGTGATCGCGTACT
**PA4843**	*gcbA*	gcbA-F	CCTGGGCACCGAATTGG
		gcbA-R	CGGCGGACAGGTAGATGATC
**PA5174**	*fabY*	PA5174-F	AGGGCGACCTGGAGATCAT
		PA5174-R	GCGCGTCCTTCTTGTATACCA
**PA5261**	*algR*	algR-F	TTGTCGATGACGAACCTCTG
		algR-R	GTCCAGTTGCCCTACCAATC

Table S3. SNPs and deletion identified in PA0724 protein sequence from dH103Pf4^{+} strain.

Effect	PAO1 position	Nucleotide change	Amino-acid change
**Missense**	791929-791930	AT>GC	Asn187Ser
**Missense**	792114	A>G	Ser249Gly
**Missense**	792124	G>A	Gly252Asp
**Codon deletion**	792190-792192	GCG>-	Gly274-
**Missense**	792401	C>A	Asp344Glu
